# Estimation of diffusion coefficients during carrots cooking in arsenious solution at different temperatures

**DOI:** 10.1016/j.heliyon.2024.e24285

**Published:** 2024-01-14

**Authors:** Oscar D. Galvez, Mariela B. Maldonado, María C. Vargas, Graciela Affranchino, Juan I. González Pacheco

**Affiliations:** a Basic Subjects Department, Mendoza Regional Faculty, National Technological University, Rodriguez 273 (M5502AJE), Mendoza City, Argentina; b CONICET, National Council for Scientific and Technical Research, Argentina; c Chemical Engineering Department, Mendoza Regional Faculty, National Technological University, Rodriguez 273 (M5502AJE), Mendoza City, Argentina

**Keywords:** *Daucus carota*, Mathematical modelling, Mass transfer phenomenon, Activation energy, Arsenic

## Abstract

This study is based on an investigation of the transport phenomenon, specifically the quantification of arsenic diffusion in carrots within a temperature range of 89 °C–99 °C using a thin plate model. Studying the diffusion of arsenic in carrots is important due to its toxicity, as it can concentrate during cooking. The World Health Organization considers arsenic as one of the ten chemical substances of public health concern. In this study, biennial hybrid carrots of the Nantesa variety were cooked whole with their epidermis in an aqueous solution containing diarsenic trioxide with an As concentration of 5 mgL^−1^ at 89 °C, 94 °C, and 99 °C. The cooking times of the carrots at different temperatures were based on a specific degree of tenderness, with a value of ≤3 kg m^−2^. The evaluated data showed consistency with increasing temperature. The calculated effective diffusion coefficients at temperatures of 89 °C, 94 °C, and 99 °C were 5.84E-09 m^2^s^-1^, 1.08E-08 m^2^s^-1^, and 2.51E-08 m^2^s^-1^ for the flesh (D_L_), and 1.601E-11 m^2^s^-1^, 2.15E-11 m^2^s^-1^, and 4.39E-11 m^2^s^-1^ for the epidermis (D_E_), respectively. The activation energy for diffusion was determined to be 159.54 kJmol^−1^ for the and 110.68 kJmol^−1^ for the epidermis. Similar behaviours were observed in different radial positions of the carrot, where the arsenic content decreased from the periphery to the centre, consistent with studies on diffusion phenomena with other solutes in food. The novelty was the detailed quantification of arsenic diffusion in the Nantes-type hybrid carrot matrix. This study is limited to a specific concentration of 5 mgL^−1^ of arsenic solution. The findings of this study may have significant implications for public health and food safety.

## 1 Introduction

Heavy metals are ubiquitous in our environment due to a variety of sources, both natural and human-induced, such as mining, industry, and agriculture. Heavy metal pollution in water resources and agricultural lands is a global problem, with toxic effects dependent on the dose and duration of exposure. Industrialization and urbanization have led to an increase in anthropogenic sources and the input of heavy metals into the environment. Heavy metals are often considered hazardous to the environment due to their toxicity, persistence, and bioaccumulative nature.

These metals originate from natural sources and human activities, being released into the air, water, and soil as a result of various activities, whether of geological or anthropogenic origin. Heavy metal pollution of water resources is a critical concern, adversely affecting plants, animals, and human health. The bioavailability and toxicity of these elements largely depend on their speciation, with soluble species being the most mobile and toxic.

Essential metallic elements are vital for biological functions in living organisms, including Mn, Fe, Co, Cu, and Zn, while non-essential metals lack a known biological function. Non-essential metals such as Cd, Pb, Hg, and As are known for their environmental hazard, and exposure to these heavy metals can have serious consequences for human health.

The trophic transfer of heavy metals in food chains is an important area of research. Both aquatic and terrestrial organisms are exposed to these heavy metals in contaminated environments. These elements transfer from the abiotic environment to living organisms, resulting in bioaccumulation and sometimes biomagnification of these elements in food chains. The transfer of these elements from soil to plants is an important route for their entry into food chains. Consuming crops contaminated with heavy metals poses a significant risk to human health. Prolonged ingestion of unsafe levels of these metals through food can lead to disruptions in biological and biochemical processes in humans. Exposure to heavy metals such as lead and mercury can cause autoimmune disorders, affecting various body systems such as joints, kidneys, the circulatory system, and the nervous system. Chronic ingestion of inorganic mercury leads to neurological and psychological effects, including tremors, personality changes, and sleep disorders. Ingesting inorganic arsenic can cause cancers at multiple sites in the human body.

The bioaccumulation of these elements in biota results in the contamination of food chains with these elements. The enrichment of these non-essential heavy metals in biota negatively affects the health of exposed organisms and their consumers. Therefore, the bioaccumulation and biomagnification of these non-essential heavy metals have significant implications for wildlife, human health, and food production [[1], [2], [3], [4], [5], [6], [7]].

When using metals as packaging material, the composition of the final product and its interactions must be considered to prevent any adverse effects on food quality and safety [8].

The practical situation of cooking food in regions with arsenic-contaminated water is an unquantified problem. Therefore, studying arsenic diffusion in carrots is important, as it is a toxic substance that can concentrate during cooking. Additionally, arsenic diffusion in food is concerning due to its potential negative effects on public health. According to the World Health Organization (WHO), inorganic arsenic is a confirmed carcinogen and is one of the most significant chemical contaminants in worldwide drinking water. Arsenic is regarded as one of the top ten most concerning chemical species [[9], [10], [11]].

Arsenic (As) is a naturally occurring element in rocks, soils and can reach groundwater through solubilization or leaching processes. Industrial activities may also contribute to increased arsenic concentrations, leading to levels above established limits for drinking water in different countries according to their regulations [[12], [13], [14]]. Plants can absorb both inorganic and organic arsenic from the soil through their roots, moreover, since in many inhospitable communities, contaminated water is an unquantified problem, the implications of these findings for public health and food security are significant. Arsenic is a toxic element that can cause a variety of health problems, including cancer, cardiovascular diseases, developmental disorders, and neurological damage. Chronic exposure to arsenic through ingestion of contaminated foods can have long-term detrimental effects. Food safety is compromised when this metalloid enters the food chain, primarily through the consumption of arsenic-laden water, the consumption of vegetables grown in contaminated soils irrigated with groundwater containing arsenic, or through the cooking of food in arsenic-contaminated water. If contaminated foods are regularly consumed, there is a health risk for the population, especially for those who heavily rely on a plant-based diet [3].

Metallic arsenic is insoluble in water, and the most common species of arsenic in water are As(III) or As(V) oxyanions. Redox potentials and pH are the most important factors controlling the speciation and mobility of the element, as well as variations in its concentration in surface water and groundwater. As(III) is the most mobile and, at the same time, the most toxic form [15].

Arsenic ingestion through drinking water over a period of time leads to a severe disease called Chronic Regional Endemic Hydroarsenicism (HACRE). This disease affects different provinces of the Argentine Republic, moreover, socially, HACRE is a disease that affects the population with limited resources in rural areas without access to a potable water supply network [[16], [17], [18], [19]].

Globally, several countries such as Bangladesh, Chile, China, Hungary, India (West Bengal), Mexico, Romania, Taiwan, Vietnam, and the southwestern region of the United States have reported arsenic concentrations exceeding 50 μg/L in large aquifers [14]. Regarding the Iberian and Ibero-American regions, arsenic contents in natural waters range from values below 0.5 μg/L to values higher than 5000 μg/L, further, in the United States, 2 % of drinking water reserves exceed 20 μg/L of arsenic [17,20,21].

In South America, the World Health Organization highlighted that four million people put their health at risk by permanently drinking water with arsenic levels above the established limits. On the one hand, in Argentina, approximately two and a half million inhabitants are at risk of suffering from diseases caused by ingesting of contaminated water with arsenic, in addition, the most affected provinces are Catamarca, Chaco, La Pampa, San Luis, San Juan, Córdoba, Jujuy, Salta, Santiago del Estero, Santa Fe, Tucumán, and Mendoza. Furthermore, in San Juan, Mendoza, La Pampa, and Neuquén, the predominant species in bodies of water is As(III), while in groundwater in Buenos Aires, Córdoba, Tucumán, and Santa Fe, the main species is As(V) [12,19,22].

On the other hand, at regions where levels of inorganic arsenic concentration in drinking water are between 50 and 100 μg/L, there is evidence of adverse effects. In other areas where arsenic concentrations in water oscillate from 10 to 50 μg/L, the World Health Organization has concluded that while there is a risk of adverse effects, the incidence may be low and difficult to detect through epidemiological studies. Consequently, the WHO established a guideline value for arsenic in drinking water worldwide as a reference for regulations and standardization in this area. Currently, the recommended limit for arsenic concentration in drinking water is 10 μg/L, given arsenic toxicological studies and the development of new analytical methods for its quantification [10,11,14].

Regarding the ingestion of contaminated food by arsenic, in Argentina, the Argentine Food Code, in its Article No. 156, incorporated the Resolution SPReI and SAGyP No. 116 and 356/2012 jointly by the Secretariat of Policies, Regulation, and Institutes (S.P.R.E.I.) and the Secretariat of Agriculture, Livestock, and Fisheries (S.A.G.Y.P.), which refers to maximum limits of inorganic contaminants in food. The maximum limit for arsenic in roots and tubers is 0.20 mg/kg.

On the other hand, in the food industry, water transport in foods is of great importance, as well as the physicochemical and microbiological changes in food substances. The transport of water to and from foods is crucial for maintaining the quality and preservation of food. Mass transfer by diffusion or passive substance transport in food systems is primarily governed by six factors, which are: Fick's law, molecular size, lipid solubility, degree of ionization, the drag effect or mass flow of water absorption, and the Donnan distribution effect, with molecular diffusion being the accepted basic transport phenomenon. The diffusion model is the one that best explains water transport, as well as the diffusion of small molecules and solutes in food, providing satisfactory results in engineering and technological processes [[23], [24], [25], [26]].

Diffusion is the process by which molecules, ions, or other small particles spontaneously mix, moving from regions of relatively high concentration to regions of lower concentration [24].

The mass transfer phenomenon has been modelled using the principles of Fick's diffusion theories, irreversible thermodynamics, multi-component diffusion, and hydrodynamic flows.

In diffusion processes, mathematical modelling serves to describe how a system works, explaining its behaviour and characteristics. It is an approximate representation that can predict, based on physical principles, the real situation of a process with reasonable accuracy [[27], [28], [29]].

Understanding the structure, size, and morphology of the food is vital for calculating solute diffusivity and transport processes. Diffusion coefficients in gases and liquids can be accurately evaluated, unlike coefficients in solids and polymers, which are abundant in food matrices. The theoretical prediction of solute diffusivity in solid and semisolid porous foods is complex, requiring measurements and experimental data [30]. It is assumed that molecular diffusion controls water transport in solids, where the driving force is a concentration gradient (dC/dz) or the equivalent moisture content gradient (dX/dz). Actually, solute diffusion in a food matrix is multidimensional, but in simplified analysis and calculations, diffusion is considered one-dimensional, and the diffusion equation of Fick's second law is applied [24,25,31].

In several studies, partial differential equations can be analytically solved based on various assumptions such as uniform initial concentration, solid geometry (flat sheet, sphere, cylinder), unidirectional material transfer, absence of contraction and expansion, zero external resistance to material transfer, and constant or variable effective diffusion coefficient [31,32].

For instance, Matusek (2002) calculated diffusion coefficients in sliced and layered carrots using sugar as a solute. Sing (2007) analysed these coefficients in cubed carrots in a ternary solution of sodium chloride, sucrose, and water. Durrani et al. (2011) did the same using honey as a solute in osmotic dehydration of carrots [[33], [34], [35]].

In general, the mass diffusion coefficient D is a parameter that depends on temperature and the concentration of components in the mixture [26]. Particularly, the arsenic diffusivity in carrots has not been quantified. Instead, it has been estimated based on the diffusivities of other non-electrolytes using various empirical formulas that relate diffusivity to molar volume [[36], [37], [38], [39]].

The present work is based on an investigation of the transport phenomenon, in which the quantification of arsenic diffusion in carrots was carried out within a temperature range of 89 °C–99 °C using a thin plate model substantiated on Fick's second law as a first approach to understand the orders of magnitude of the effective diffusion coefficients in order to characterize the process.

## 2 Materials

Biennial hybrid carrots of the Nantesa varietal type obtained from a carrot producer in Mendoza, Argentina, were used. A batch of 220 selected carrot samples with a truncated cone shape was sampled. The average dimensions of the carrots were: length 179.64 ± 1.63 mm; weight 152 ± 3.74 g; upper, middle, and lower diameters of 37.4 ± 0.51 mm, 32.1 ± 0.39 mm, and 26.7 ± 0.48 mm, respectively.

Diameter and height measurements of the carrots were taken using a 0–200 mm digital calliper (Ruhlmann brand) and a 30 cm metal ruler (Brand brand), respectively. Weights were measured using a digital balance (Ohaus brand, model SP601) with a sensitivity of 0.1 g.

The diameter readings were taken in triplicate with each carrot rotated axially by 120° in three regions of the vegetable sample: upper (2 cm below the stem), lower (3 cm above the root tip or lower end), and middle (halfway along the length of the sample). As carrots are biological material, it was difficult to determine a priori the diffusion of arsenic within the matrix. Therefore, as an arbitrary method and not knowing any other reference method, the degree of tenderness was chosen as the endpoint of the assay.

## 3 Methods

By establishing the degree of tenderness in the carrot, we assumed equal final cooking conditions. Three temperature levels were used, with a range of 5 °C between them.

The whole carrots with their epidermis intact were cooked in distilled water at the following temperatures: T1 = 99 °C ± 1 °C, T2 = 94 °C ± 1 °C, and T3 = 89 °C ± 1 °C, in a thermostatic bath (Technicon brand, Autoanalyzer II model) with agitation at 1150 rpm.

The cooking time or duration of the assay at different temperatures was determined based on a specific value of tenderness in the three regions: upper, middle, and lower. The target value was equal to or less than 3.0 kg m^−2^ [40]. Measurements were taken on 85 carrots from the initial batch of 220 carrots. The cooking times at different temperatures along with the tenderness values are shown in Table 1.

**Table 1:** Cooking times at different temperatures.

Temperature (°C)			Degree of tenderness (kg.m^−2^)	
	Time (minutes)	Region	Mean	SD
99	30	Upper	2.8	0.52
		Middle	2.2	0.41
		Lower	1.8	0.45
94	60	Upper	3.0	0.39
		Middle	2.7	0.47
		Lower	2.2	0.42
89	240	Upper	3.0	0.19
		Middle	2.7	0.25
		Lower	2.4	0.33

Once the cooking time was defined for each assay, the sampling interval was set to be one-fourth of that time. Subsequently, to measure the progress of arsenic in the carrot over time, the cooking of whole carrots with their epidermis was carried out in the same thermostatic equipment with continuous agitation throughout the assay, at different temperatures using an arsenic (As) solution with a concentration of 5 mgL^−1^. The zero time point was considered for the carrots just before immersing them in the arsenical solution at the test temperature. The proposed arsenic concentration value is in this study high to fall within the detection range of the employed equipment. The technology used limited the range of useable concentrations, hence the solution employed was a model solution to determine the value of a parameter, such as the diffusion coefficient (D) for arsenic in carrots, which has not yet been directly quantified in the reviewed publications but is important for understanding the behaviour of the phenomenon and quantifying the process. The As solution for the cooking of carrots was prepared from a stock solution of 1000 mg/L arsenic, which contained 1.320 g of arsenic trioxide, As_2_O_3_ (analytical grade) and 4.000 g of NaOH, then diluted with distilled water (resistivity 100 mOhms) to 1 L [41]. The solubility of arsenic trioxide in water is 1.2–3.7 g/100 mL of water at 20 °C.

The manuscript underwent modifications in paragraphs, and in the Methodology section, the control test and purpose were mentioned.

Since an arsenic model solution was used to determine the diffusion coefficient and to ensure that the arsenic particles found in the plant tissue were the result of arsenic diffusion from the model solution, the arsenic content in the carrot was quantified before commencing the experiment. The control test for arsenic determination was conducted on 27 raw carrots from a batch of 220 carrots. The measured average value was 0.127 μg g^−1^ of arsenic. This found value was subtracted from the quantified arsenic content in all the samples cooked at different temperatures.

For the rest of the carrots, in each assay, 12 carrots were used, and for each carrot, 9 samples were obtained for subsequent determination of total arsenic.

Prior to immersing and cooking of the carrots in the arsenic solution, the ends were coated with a waterproof coating (asphalt paint) to prevent longitudinal solute transfer during cooking. The total arsenic content in cooked carrots were evaluated based on the concentration of the arsenic solution during cooking, while maintaining a constant temperature. Three (3) carrots were extracted at each time interval.

**Fig. 1:**
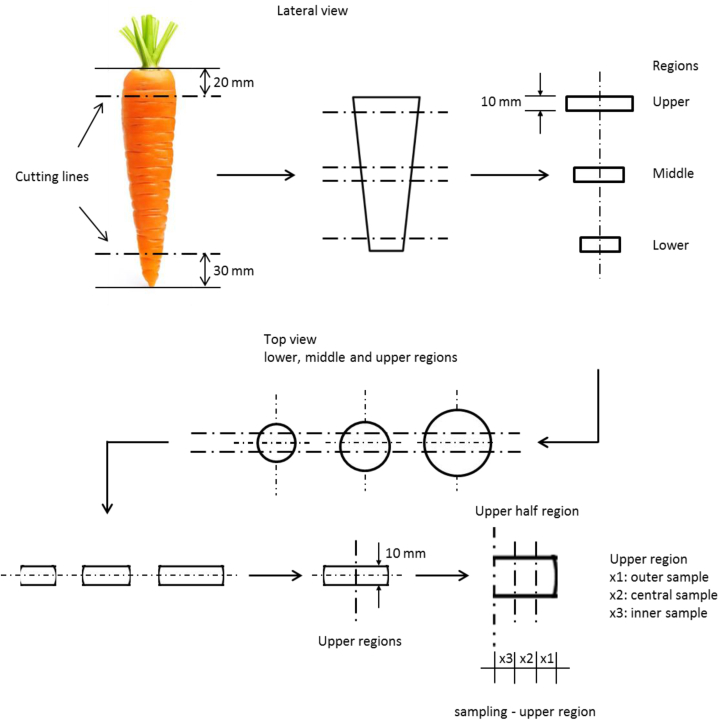
Sequence of cuts for obtaining samples.

The raw and cooked carrots underwent a series of cuts to obtain samples based on the cut height (carrot length) and radial distance of the sample. Fig. 1 shows the sequence of cuts for the upper cut sample, and the same procedure was carried out for the samples from the middle and lower regions. After the test, acid treatment and As quantification by AAS were carried out on the samples [41].

The coding used in the figure legends corresponds to a sample unit (×1, ×2, or ×3) from a sample region (upper, middle, or lower) of the carrot. For example, the legend “×1” refers to the portion of radial distance near the outer part of the carrot flesh in a specific region, as detailed in Fig. 1.

### 3.1 Statistical analysis

All statistical analyses were performed using GraphPad Prism 8, version 8.0.1.

### 3.2 Mathematical modelling

Considering the sample's geometric shape, a thin plate model was proposed. Following the experiment development, it was required to use the following assumptions.

1. Molecular diffusion is the only transport mechanism within the solid, thus convective transport is neglected (v_x_ = v_y_ = v_z_ = 0).
2. No generation of substances by chemical reaction is considered.
3. Carrot flesh is considered homogeneous and its properties isotropic.
4. Molecular diffusion is unidirectional.
5. Due to great agitation of the liquid surrounding the surfaces (elevated mass Biot number), the concentration in epidermis instantly acquires the liquid concentration.

$$C_{i}\left(x_{i},t\right)=C_{i\left(x\;\sup\right)}=C_{soluc}\;;\;t>0$$

6. Isothermal process is considered.
7. Usually, in the phenomenon of diffusion in food, food systems are considered as porous solids [30]. The thickness of the epidermis is negligible and difficult to separate operationally using simple techniques without dragging part of the mesocarp, so the value from the literature was assumed. However, it could present a barrier phenomenon to solute diffusion, which is common in most studies [37,38,[42], [43], [44], [45], [46]].

**Fig. 2:**
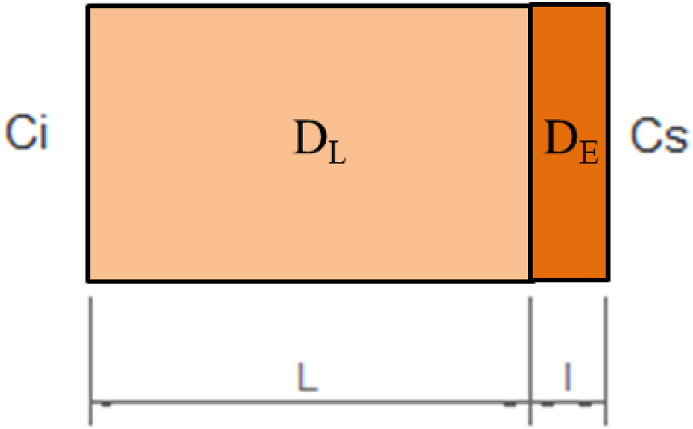
Thin plate model.

Fig. 2 illustrates the simplified thin plate model for the upper cut sample.

Where:

Cs: concentration of arsenic on the external surface of the carrot in [μg/g As].

Ci: initial concentration of arsenic in the sample in [μg/g As].

D_L_: diffusion coefficient of the flesh in [m^2^/s].

D_E_: diffusion coefficient of the epidermis in [m^2^/s].

L: length of flesh in the upper, middle, and lower regions of the batch of 220 carrots in [m].

L_upper_ = 18.70E-03 m.

L_middle_ = 16.05E-03 m.

L_lower_ = 13.35E-03 m.

l = 8.80E-06 m. Thickness of the epidermis obtained from Escobar et al. (2017) [47].

The experimental approach used in this study was a variation of the methods applied by Crank (1975) and Maldonado (2003) [31,42].

Limitations of the method used:

• A thin plate model was considered when actually the samples come from a cylinder.
• The flesh was assumed to be isotropic and homogeneous, however it has different morphological configurations. To solve the diffusion model, various publications consulted use the resolution of Fick's second law, which determines a diffusion coefficient D for the flesh and allows the calculation of a coefficient D for the epidermis. However, to solve these equations, isotropy and homogeneity are assumed (as if there were no differences). The reality is that the epidermis in most vegetables has a different composition from the flesh due to physiological and functional characteristics that are not the focus and detail of this study. These differences that exist are what later allow the interpretation of results when the values of flesh and skin D are differential.
• It is applied within a certain range of temperature and arsenic concentration.

For the one-dimensional thin plate model, Fick's second law of diffusion, Equation (1), is subject to the following initial and boundary conditions, Equation (2-a), (2-b), (2-c).

(1) $$\frac{\partial C}{\partial t}=D_{L}\frac{\partial^{2}C}{\partial x^{2}}$$

(2-a) $$\text{CI}:C_{\left(x,t\right)}=C_{i};\;0\leq x\leq L;\;t=0$$

(2-b) $$\text{CF}1:\frac{\partial C}{\partial x}=0;\;x=0;\;t>0$$

(2-c) $$\text{CF}2:\frac{\partial C}{\partial x}=-\frac{D_{E}}{D_{L}}\left(\frac{C_{\left(x,t\right)}-C_{s}}{l}\right);\;x=L;\;t>0$$

where the dimensionless variables of Equation (1) are given by Equation (3):

(3) $$\tilde{C}=\frac{C_{\left(x,t\right)}-C_{s}}{C_{i}-C_{s}};\;\tilde{X}=\frac{x}{L};\;\theta =t\frac{D_{L}}{L^{2}}$$

(4) $$\begin{array}{l} \frac{\partial \tilde{C}}{\partial \theta }=\frac{\partial^{2}\tilde{C}}{\partial {\tilde{X}}^{2}}\; \end{array}$$

The dimensionless Equation (4) is the equation for the one-dimensional diffusion process with constant effective diffusion coefficients in flesh (D_L_) and epidermis (D_E_).

Therefore, the initial and boundary conditions are as follows

(5-a) $$\begin{array}{l} \text{CI}:\tilde{C}=1;\;0\leq \tilde{X}\leq 1;\;\theta =0\; \end{array}$$

(5-b) $$\text{CF}1:\frac{\partial \tilde{C}}{\partial \tilde{X}}=0;\;\tilde{X}=0;\;\theta >0$$

(5-c) $$\text{CF}2:\frac{\partial \tilde{C}}{\partial \tilde{X}}=-\frac{D_{E}/l}{D_{L}/L}\tilde{C};\;\tilde{X}=1;\;\theta >0$$

The solution of Equation (4) for thin plate subjected to the conditions of Equation (5-a), (5-b), (5-c) is

(6) $$\tilde{C}\left(\tilde{X},\theta \right)=\left[1+\sum_{n=1}^{\infty }2\frac{sen\left(\lambda_{n}\right)}{\lambda_{n}}\cos\left(\lambda_{n}\tilde{X}\right)\right]\exp\left(-\lambda^{2}\theta \right)$$

The eigenvalues of λ_n_ were obtained by means of the following eigenfunction

(7) $$\lambda_{n}\;\tan\left(\lambda_{n}\right)=\frac{D_{E}}{D_{L}}\frac{L}{l}$$

The average volumetric concentration, Equation (8) is obtained by integrating Equation (6), thus

(8) $$\tilde{C}v_{m}=\frac{1}{V}\int_{V}\tilde{C}dV$$

where the thin plate dimensions are.

Length (x-axis), L, total length of the upper, middle, and lower samples in cm.

Width (y-axis), a, 1 cm.

Height (z-axis), h, 1 cm.

With regard to integration extremes, this extends from zero to one according to the conditions of Equation (5-a), hence

$$\begin{array}{l} \tilde{C}v_{m}=\frac{1}{V}∭VdV=\frac{1}{V}\int_{0}^{L}\int_{0}^{a}\int_{0}^{h}Cdxdydz\\ \tilde{C}v_{m}=\frac{1}{L\times a\times h}\int_{0}^{L}\left\{\left[1+\sum_{n=1}^{\infty }2\frac{sen\left(\lambda_{n}\right)}{\lambda_{n}}\cos\left(\lambda_{n}\frac{x}{L}\right)\right]\exp\left(-\lambda^{2}D_{L}t/L^{2}\right)\right\}dx\int_{0}^{a}dy\int_{0}^{h}dz \end{array}$$

(9) $$\tilde{C}v_{m}=\left[1+2\sum_{n=1}^{\infty }\frac{sen^{2}\left(\lambda_{n}\right)}{\lambda_{n}^{2}}\right]\exp\left(-\frac{\lambda_{n}^{2}D_{L}t}{L^{2}}\right)$$

for long process times, the terms for n > 1 in Equation (9) have scarce contribution, consequently the series converges in the first term (n = 1), the equation can be reduced to

(10) $$\tilde{C}v_{m}=\left(\frac{1+2sen^{2}\left(\lambda \right)}{\lambda^{2}}\right)\exp\left(-\frac{\lambda^{2}D_{L}t}{L^{2}}\right)$$

The diffusion constant of the flesh and the eigenvalues for each experiment were estimated using the Least Squares method, which minimizes the equation:

(11) $$S={\sum_{i=1}^{N}\left({\tilde{C}}_{vm\;\exp.}-{\tilde{C}}_{vmcalc.}\right)}^{2}$$

where ${\tilde{C}}_{vm\;\exp.}$ is the experimental volumetric concentration and ${\tilde{C}}_{vmcalc.}$ is the volumetric concentration estimated by calculating Equation (10). Since the sum of the squared residuals is given by Equation (11) and is a nonlinear function of the effective diffusion coefficient of the carrot flesh and the eigenvalues, the nonlinear regression method of the Microsoft Excel Solver® was used to calculate the value of the skin diffusivity (D_E_) using Equation (7). The diffusion coefficient of the epidermis was calculated using Equation (7). The value of the epidermis thickness (l) was obtained from (Escobar-Avila et al., 2017). The quantified diffusivities can be modelled as a function of temperature using the Arrhenius dependence, Equation (12).

(12) $$D_{L}=D_{0}\;\exp\left(-\frac{E_{a}}{R.T}\right)$$

The applied mathematical model was used to fit the data of the effective diffusion coefficients at different temperatures using linear regression.

For more information about the mathematical development and results, please refer to Supplementary Material.

## 4 Results and discussions

### 4.1 Experimental part

#### 4.1.1 Arsenic concentration profiles

**Fig. 3:**
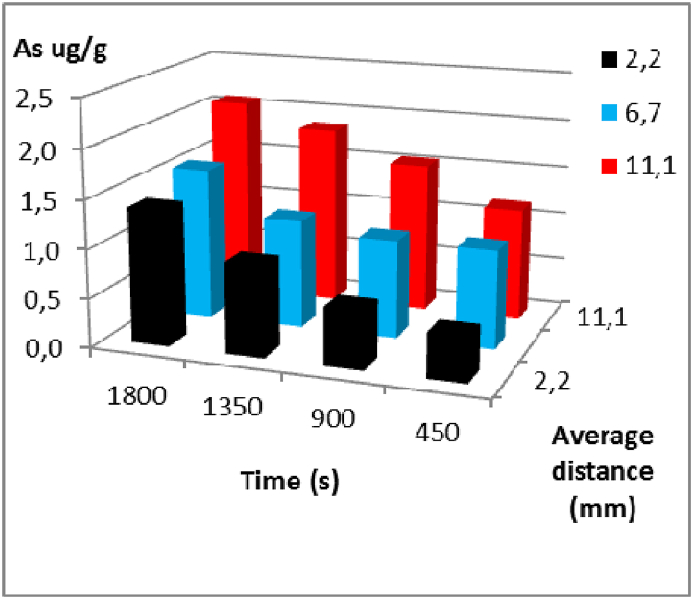
Arsenic concentration versus cooking times for each average distance from the centre of the carrot to the epidermis in the lower region. Experiment at 99 °C with a 5 mg/L As solution respectively.

**Fig. 4:**
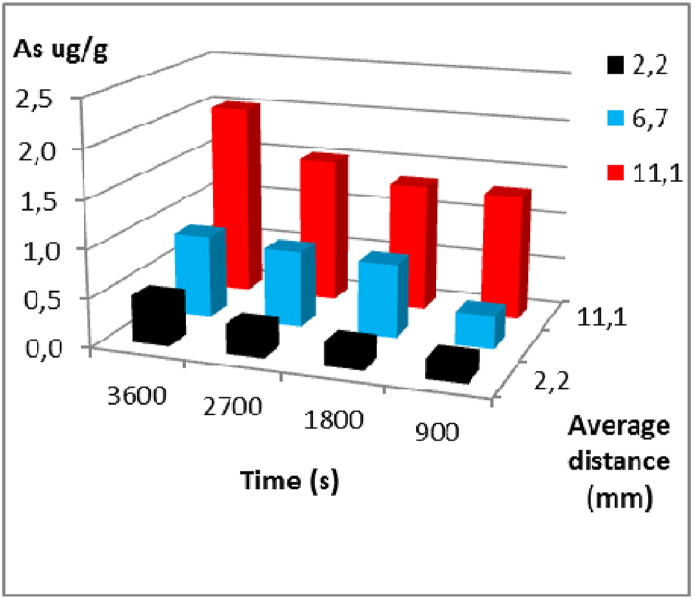
Arsenic concentration versus cooking times for each average distance from the centre of the carrot to the epidermis in the lower region. Experiment at 94 °C with a 5 mg/L As solution respectively.

**Fig. 5:**
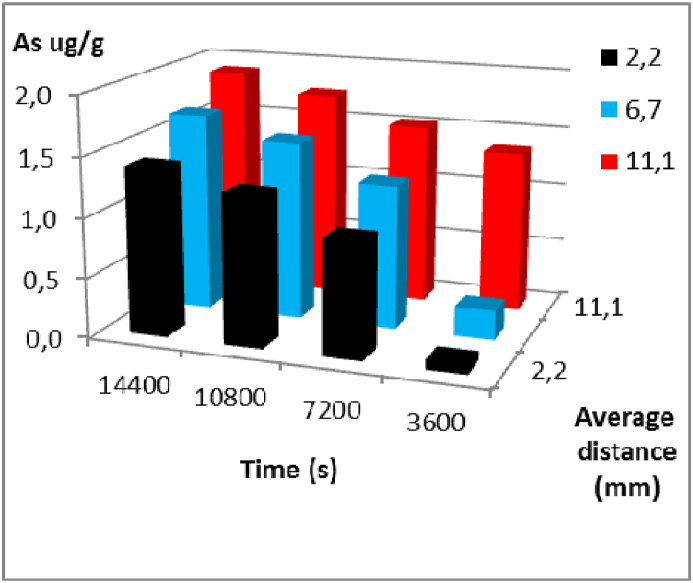
Arsenic concentration versus cooking times for each average distance from the centre of the carrot to the epidermis in the lower region. Experiment at 89 °C with a 5 mg/L As solution.

The arsenic concentration profiles during the cooking of carrots with a 5 mg/L As solution are shown in Fig. 3, Fig. 4, Fig. 5 for experiments at temperatures of 99 °C, 94 °C, and 89 °C, respectively, for the lower regions or cuts. The recorded distances represent the midpoint of the cuts, measured from the centre of the carrot towards the epidermis.

The concentration profiles of arsenic in the lower region are illustrated since it is the area where the diffusion phenomenon in carrots is most evident. As observed in Fig. 3, Fig. 4, Fig. 5, the concentration of arsenic decreases as it diffuses through the carrot from the epidermis towards the centre. For example, in the first assay, at 30 min, at a depth of 11.1 mm (a sample section that includes the epidermis, ×1), the concentration of arsenic was 2.081 μg g^−1^ As, at 6.7 mm (an intermediate sample section between the epidermis and the centre of the carrot, ×2), it was 1.564 μg g^-1^ As, and at 2.2 mm (near the centre, ×3), it was 1.388 μg g^-1^ As. This pattern is observed in each sampling interval and region (middle and upper) for all assays conducted at different temperatures.

The figures also demonstrate that, with regard to the time variable, as the assay duration increases, more arsenic enters the carrot matrix, assuming a constant concentration gradient, except in the cuts ×2 and ×3, where concentrations are higher at 99 °C and 89 °C than at 94 °C. This could be due to being a biological material, carrots may exhibit frequent variations in the nature of the matrix under study.

Generally, the concentrations for different distances and sampling intervals are higher as the temperature increases, at equal time intervals.

The total amount of arsenic in vegetables can be reduced through pre-cooking, peeling, washing, and cutting treatments. Vegetables grown in contaminated soil or irrigated with contaminated water generally accumulate more inorganic As [48].

Devesa et al. (2008) examined the levels of inorganic arsenic in vegetables and cereals using water with varying concentrations of arsenic. The results showed that the amount of inorganic arsenic varied in some products, depending on the type of water used. In products cooked with water containing high levels of inorganic arsenic, an increase in this form of arsenic was observed compared to the raw product. On the other hand, foods cooked with distilled water presented lower levels of inorganic arsenic compared to those detected in the food before cooking, and when vegetables are cooked in arsenic-boiling water, as occurs in arsenic-endemic regions, the resulting food has high concentrations of inorganic arsenic. After cooking, vegetables mainly containing inorganic arsenic (iAs) show scarse variability, due to the high stability of these forms of arsenic [49]. The data showed that decreasing the temperature reduces the amount of arsenic entering the carrot matrix, further this reduction occurs from the outside to the inside, as well as the progressive relationship between the total amount of diffused substance and time. Similar behaviours were found by M. Maldonado et al. (2003) for sodium diffusion in olives [42]. Chilev et al. (2014) worked with water-ethanol at different temperatures in the solid-liquid extraction of Cotinus coggygria [50]. Kusnadi et al. (2012) analysed the diffusion of sodium salt in celery, mushrooms, and chestnuts over a temperature range of 25–80 °C [51]. An inverse analogy to that analysed by Della Rocca et al. (2014) was also highlighted in the moisture removal from carrot cubes with sucrose and sodium chloride at 40 °C, as well as in rectangular pieces of peaches and carrot cubes at different temperatures in Refs. [36,39,52]. It is known that decreasing the temperature of the cooking solution also decreases the mean square velocity of solute particles and thus kinetic energy, leading to a decrease in the driving force of the substance diffusing through the carrot flesh. Acording this data, the behaviour of diffusion laws was verified as a function of temperature, where an increase in the thermal gradient leads to a greater amount of diffused solute in the radial distances of the carrot, at equal measurement times [25,31,53].

Table 2 presents the activation energy values and the effective diffusion coefficients of arsenic for the upper, middle, and lower regions in the skin and flesh of carrots for the three treatments with an arsenic concentration of 5 mg/L.

**Table 2:** Average effective diffusion coefficients and Activation Energy in thin plate model.

Temperature		Effective diffusion coefficients (m^2^s^−1^)			
		Region			
		Upper	Middle	Lower	Average
89 °C	D_L_	5,34E-09	5,55E-09	6,64E-09	**5,84E-09**
	Std. Error	1,80E-09	1,29E-09	3,73E-09	
	D_E_	4,71E-12	1,25E-11	3,07E-11	**1,60E-11**
	R^2^	0,938	0,921	0,780	
94 °C	D_L_	1,07E-08	1,06E-08	1,10E-08	**1,08E-08**
	Std. Error	7,32E-10	8,90E-10	4,13E-10	
	D_E_	7,24E-12	1,21E-11	4,51E-11	**2,15E-11**
	R^2^	0,988	0,965	0,982	
99 °C	D_L_	8,06E-09	1,76E-08	4,95E-08	**2,51E-08**
	Std. Error	2,17E-05	1,53E-08	1,07E-09	
	D_E_	4,85E-15	6,30E-12	1,25E-10	**4,39E-11**
	R^2^	0,943	0,943	0,999	
	Ea	D_0_	R^2^		
	(KJmol^−1^)	(m^2^s^−1^)			
Flesh	**159.54**	1.86E+15	0.990		
Epidermis	**110.68**	3.09E+05	0.943		

From the analysis of Tables 2 and it was observed that the values of D_L_ and D_E_ decreased as the temperature decreased for a constant concentration of arsenic in the solution during carrot cooking.

With a temperature decrease of five degrees between trials a 99 °C and 94 °C, and ten degrees compared to trials 99 °C and 89 °C, the percentage decrease of the effective diffusion coefficients in carrot flesh (D_L_) was 56.1 % and 77.5 % respectively. Additionally, the effective diffusion coefficients for different temperatures were significantly different, indicating that temperature directly affects these coefficients. In other words, at lower temperatures, the rate of advancement of arsenic molecules decreases, which was also corroborated by Maldonado et al. (2003) [42]. A similar conclusion was verified by Kusnadi et al. (2012), where the diffusion coefficients of sodium salt were significantly affected by temperature in vegetable tissues such as celery, mushrooms, and chestnuts [51].

The low values of the diffusion coefficient in the epidermis (D_E_) indicate that this external surface generated resistance to solute transfer from the solution to the internal parts of the carrot. This is consistent with the findings of Maldonado et al. (2003) [42].

The arsenic diffusivity values found in carrots ranged from 5.53E-09 to 2.46E-08 m^2^s^-1^, which are consistent with the findings of Melquiades et al. (2009) in the hydration or rehydration processes of peeled and sliced carrots, which ranged from 3.46E-10 to 4.59E-10 m^2^s^-1^ in the temperature range of 40–80 °C [37].

The results obtained in this study are limited to a specific concentration of 5 mgL^−1^ of arsenic solution, as the diffusion coefficient and activation energy change with concentration.

On the other hand, Zambrano et al. (2007) reported water diffusion coefficients ranging from 6.11E-09 to 3.18E-09 m^2^s^-1^ at temperatures between 50 and 93 °C for freeze-dried carrots. Della Rocca et al. (2014) found an effective water diffusion coefficient value of 1.57E-9 m^2^s^-1^ through osmodehydrofreezing. Both values are consistent with those found in this study [36,38].

The diffusion coefficients reported in Table 2 are also consistent with Saravacos and Maroulis (2001), who presented typical values of moisture diffusivity for various food products at different temperatures, including carrots: 2.00E-10 m^2^s^-1^ at 30 °C, 2.20E-12 to 7.46E-09 m^2^s^-1^ for 20 °C and 100 °C [25].

Furthermore, these values are lower than those obtained by Matusek et al. (2002) for sugar diffusion in carrots, with values on the order of 1.00E-11 m^2^s^-1^, consistent with the molecular mass of sugars greater than arsenic [34].

## 5 Conclusions

The diffusion of arsenic in the Nantes-type hybrid carrot matrix was quantified.

The diffusion phenomenon of arsenic in carrots was quantified in the temperature range between 89 °C and 99 °C. At the same temperature, similar behaviours were observed in all regions: upper, middle, and lower, where arsenic content decreased from the periphery towards the centre of the carrot, consistent with other studies on diffusion phenomena with other solutes in food. Due to the conical shape of the carrot, it was demonstrated that at a shorter radial distance, specifically in the lower region, the advancement of arsenic in the carrot increased. The average effective diffusion coefficients calculated at temperatures of 89 °C, 94 °C, and 99 °C were 5.84E-09 m^2^s^-1^, 1.08E-08 m^2^s^-1^, and 2.51E-08 m^2^s^-1^ for the flesh (D_L_), and 1.60E-11 m^2^s^-1^, 2.15E-11 m^2^s^-1^, and 4.39E-11 m^2^s^-1^ for the epidermis (D_E_), respectively. The temperature dependency of these coefficients was verified through the Arrhenius equation, which yielded an activation energy of 159.54 kJmol^−1^ for the flesh and 110.68 kJmol^−1^ for the epidermis. The low values of the diffusion coefficient in the epidermis (D_E_) indicated that this external surface generated resistance to solute transfer from the solution to the internal parts of the carrot. This study is limited to a specific concentration, which is 5 mgL^−1^ of arsenic solution, as the diffusion coefficient and activation energy change with concentration. The arsenic values diffused in the carrots in the experiments exceeded the maximum limit of inorganic As in food (0.2 mgKg^−1^), according to SPReI and SAGyP Resolution No. 116 and 356/2012, which means that the findings may have significant implications for public health and food safety. In the future, it is proposed to simulate the data using a solid composite cylindrical model.

## Data availability statement

Data will be made available on request.

## CRediT authorship contribution statement

**Oscar D. Galvez:** Writing – original draft. **Mariela B. Maldonado:** Validation, Supervision, Methodology. **María C. Vargas:** Supervision, Formal analysis, Conceptualization. **Graciela Affranchino:** Formal analysis. **Juan I. González Pacheco:** Writing – review & editing, Visualization, Conceptualization.

## Declaration of competing interest

The authors declare that they have no known competing financial interests or personal relationships that could have appeared to influence the work reported in this paper.

## Nomenclature

C: arsenic concentration [μg/g As]
θ: dimensionless time.
x: spatial variable [m]
l: thickness of the epidermis in [m]
t: temporary variable. Diffusion time [s]
$\lambda_{n}$: eigenvalues
D_L_: effective flesh diffusion coefficient in [m^2^s^−1^]
$\tilde{C}v_{m}$: volumetric average concentration of dimensionless arsenic
C_(x,t)_: arsenic concentration as a function of position and time [μg/g As]
V: sample volume [cm^3^]
Ci: arsenic initial concentration in the sample in [μg/g As]
a: sample width [cm]
L: average flesh length in [m]
h: sample height [cm]
Cs: arsenic concentration on the external surface of the carrot in [μg/g As]
D_0_: pre-exponential constant [m^2^s^−1^]
D_E_: effective diffusion coefficient in epidermis [m^2^s^−1^]
Ea: activation energy [KJmoL^−1^]
$\tilde{C}$: dimensionless arsenic concentration
R: universal gas constant [JmoL^−1^K^−1^]
$\tilde{X}$: dimensionless length
T: absolute temperature [K]
